# Evaluating the efficacy and safety of acotiamide in patients with esophagogastric junction outflow obstruction: study protocol for an investigator-initiated, multi-center, randomized, double-blind, placebo-controlled phase II trial

**DOI:** 10.1186/s13063-023-07468-w

**Published:** 2023-07-18

**Authors:** Mitsuru Esaki, Eikichi Ihara, Noriaki Manabe, Noriyuki Kawami, Katsuhiko Iwakiri, Junichi Akiyama, Shiko Kuribayashi, Toshio Uraoka, Haruei Ogino, Takatoshi Chinen, Akiko Misumi, Hiroko Watanabe, Maya Suzuki, Junji Kishimoto, Yoshihiro Ogawa

**Affiliations:** 1 Department of Medicine and Bioregulatory Science, Graduate School of Medical Sciences, Kyushu University, Fukuoka, Japan; 2 Department of Gastroenterology and Metabolism, Graduate School of Medical Sciences, Kyushu University, Fukuoka, Japan; 3 Division of Endoscopy and Ultrasonography, Department of Clinical Pathology and Laboratory Medicine, Kawasaki Medical School, Kurashiki, Japan; 4 Department of Gastroenterology, Nippon Medical School, Graduate School of Medicine, Bunkyo-ku, Tokyo Japan; 5 Division of Gastroenterology and Hepatology, National Center for Global Health and Medicine, Shinjuku-ku, Tokyo Japan; 6 Department of Gastroenterology and Hepatology, Gunma University Graduate School of Medicine, Maebashi, Japan; 7 Center for Clinical and Translational Research, Kyushu University Hospital, Fukuoka, Japan

**Keywords:** Esophagogastric junction outflow obstruction, Acotiamide, Esophageal motility disorders, Achalasia

## Abstract

**Background:**

We have determined that the impaired accommodation of the lower esophageal sphincter (LES) underlies the pathogenesis of esophagogastric junction outflow obstruction (EGJOO). We have also found that acotiamide may treat EGJOO by improving impaired LES accommodation. The effects of acotiamide in patients with EGJOO need to be further confirmed in a prospective study.

**Methods:**

This trial is a multicenter, randomized, double-blind, placebo-controlled study to compare the efficacy and safety of acotiamide (300 mg/day or 600 mg/day) with those of a placebo in the treatment of patients with EGJOO. The primary endpoint will be the proportion of patients who report an improvement in symptom of food sticking in the chest after 4 weeks of treatment period 1. The secondary endpoints will be the proportion of patients with normalized integrated relaxation pressure (IRP), the value of change from baseline in the distal contractile integral, basal LES pressure, EGJOO–quality of life score, Gastrointestinal Symptom Rating Scale, and the correlation between IRP and each symptom score. During the 2-year trial period, 42 patients from five institutions will be enrolled.

**Discussion:**

This trial will provide evidence to clarify the efficacy and safety of acotiamide as a treatment for patients with EGJOO. Acotiamide might help improve the quality of life of patients with EGJOO and is expected to prevent the progression of EGJOO to achalasia.

**Trial registration:**

This study was approved by the Institutional Review Board (IRB) of Kyushu University Hospital as well as the local IRBs of the participating sites for clinical trials and registered in the Japan Registry of Clinical Trials (jRCT: 2071210072). The registration date is on October 11, 2021.

**Supplementary Information:**

The online version contains supplementary material available at 10.1186/s13063-023-07468-w.

## Strengths and limitations of this study

This is the first prospective and randomized controlled trial to investigate the superiority of acotiamide over a placebo in the treatment of patients with EGJOO.

The trial provides evidence that acotiamide can be used as a treatment for patients with EGJOO.

Long-term outcomes cannot be assessed because this trial focuses on the short-term outcomes of acotiamide for the treatment of patients with EGJOO.

**Supplementary Information:**

The online version contains supplementary material available at 10.1186/s13063-023-07468-w.

## Administrative information

**Table:** 

**Title {1}**	Evaluating the efficacy and safety of acotiamide in patients with esophagogastric junction outflow obstruction: study protocol for an investigator-initiated, multi-center, randomized, double-blind, placebo-controlled phase II trial.
**Trial registration {2a and 2b}**	This study was registered in the Japan Registry of Clinical Trials (jRCT: 2071210072) on October 11, 2021.
**Protocol version {3}**	This is version 4.0 of the protocol; April 22, 2022.
**Funding {4}**	This study is funded by the Japan Agency for Medical Research and Development (AMED) (21lk0201144h001, 22lk0201144h002, and 23lk0201144h003). Investigational drugs, including acotiamide and a placebo, will be provided free of charge by Zeria Pharmaceutical Co. Ltd.
**Author details {5a}**	ME, EI, HO, TC, YO: Department of Medicine and Bioregulatory Science, Graduate School of Medical Sciences, Kyushu University EI: Department of Gastroenterology and Metabolism, Graduate School of Medical Sciences, Kyushu University NM: Division of Endoscopy and Ultrasonography, Department of Clinical Pathology and Laboratory Medicine, Kawasaki Medical School NK, KI: Department of Gastroenterology, Nippon Medical School, Graduate School of Medicine JA: Division of Gastroenterology and Hepatology, National Center for Global Health and Medicine SK, TU: Department of Gastroenterology and Hepatology Gunma University Graduate School of Medicine AM, HW, MS, JK: Center for Clinical and Translational Research, Kyushu University Hospital
**Name and contact information for the rial sponsor{5b}**	This study is investigator-initiated Principal investigator representative: Eikichi Ihara: ihara.eikichi.167@m.kyushu-u.ac.jp AMED: rinsho-kakushin@amed.go.jp
**Role of sponsor {5c}**	Although Zeria provides investigational drugs free of charge and information on the investigational drugs and their safety, Zeria is not involved in the study design, data collection, analysis, or interpretation of data in this trial.

## Introduction

### Background and rationale {6a}

Esophageal motility disorders (EMDs), which include esophageal achalasia, reduce quality of life (QOL) and social labor productivity due to dysphagia and non-cardiac chest pain and threaten life due to aspiration pneumonia in severe cases. High-resolution manometry (HRM), together with the Chicago Classification, has dramatically improved medical treatment for patients with EMDs [1, 2]. Esophagogastric junction outflow obstruction (EGJOO) is a relatively new EMD. EGJOO is characterized by impaired lower esophageal sphincter (LES) relaxation but by intact esophageal body peristalsis, whereas achalasia is characterized by impaired LES relaxation and esophageal body peristalsis. Basic and clinical studies have indicated that EGJOO is a variant or precursor of esophageal achalasia [3]. Importantly, the QOL of patients with EGJOO could be as low as that of achalasia patients owing to dysphagia and noncardiac chest pain. To date, no definitive treatment has been developed for EGJOO. The development of a fundamental treatment for EGJOO is important because problematic symptoms in patients with EGJOO can be improved and the progression of EGJOO to achalasia might also be retarded.

The LES maintains myogenic contractile pressure during the non-eating state to suppress the reflux of gastric contents into the esophagus and pharynx. During swallowing, a rapid and sufficient LES relaxation response is required to transport the bolus to the stomach. We have recently shown that successful LES relaxation is achieved by LES accommodation in combination with swallow-induced LES relaxation [4]. LES accommodation is mainly caused by the physical stimulation of the oral cavity and/or pharynx by liquid and food before the swallowing action occurs [5]. In contrast, swallow-induced LES relaxation results from the swallowing action itself. We have also found that the impairment of LES accommodation but not swallow-induced LES relaxation underlies the pathogenesis of EGJOO, where LES accommodation can be the target for the development of a fundamental treatment for EGJOO.

Acotiamide hydrochloride (acotiamide) (Zeria Pharmaceutical Co. Ltd) has been developed as a gastrointestinal acetylcholinesterase inhibitor [6] and is approved for the treatment of functional dyspepsia (FD) in Japan in 2013 [7]. Acotiamide increases the amount of acetylcholine at the cholinergic nerve ending, which improves the impaired accommodation of the gastric body and fundus and the hypomotility of the gastric antrum in patients with FD. As LES accommodation is induced similarly to gastric accommodation, we hypothesize that acotiamide can be an ideal treatment for patients with EGJOO via the improvement of impaired LES accommodation. Indeed, retrospective and prospective observational clinical studies conducted by the authors have successfully demonstrated the possibility of drug repositioning of acotiamide for EGJOO [8, 9]. However, the efficacy and safety of acotiamide in the treatment of patients with EGJOO need to be further confirmed in a prospective study.

### Objectives {7}

The trial aims to evaluate the effectiveness and safety of acotiamide in the treatment of patients with EGJOO, including the dose-response to acotiamide and a placebo. The hypothesis for the primary endpoint is that acotiamide will improve the symptoms of food sticking in the chest in patients with EGJOO after 4 weeks of treatment compared with a placebo.

### Trial design {8}

The study is an investigator-initiated, multicenter, phase II, double-blind, randomized, placebo-controlled trial with three study arms: acotiamide 300 mg/day, acotiamide 600 mg/day, or a placebo, randomized 1:1:1 and stratified by the institution.

## Methods: participants, interventions, and outcomes

### Study setting {9}

**Fig. 1:**
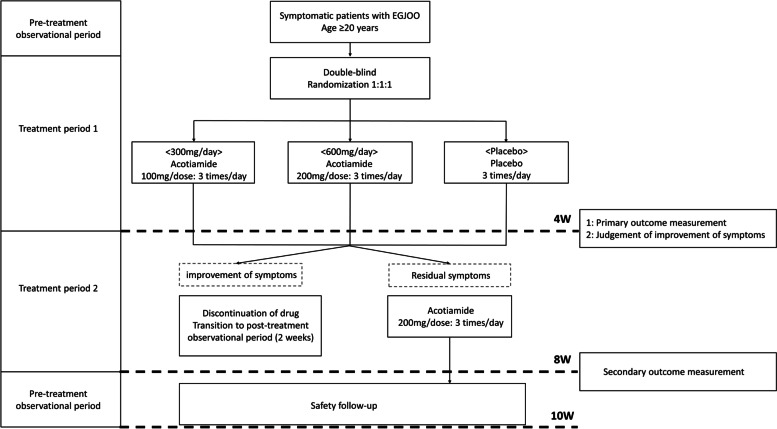
Schematic of the study design. EGJOO, esophagogastric junction outflow obstruction

This study will be conducted at five Japanese institutions with experts in the treatment of EMDs: Kyushu University, Kawasaki Medical School, Nippon Medical School, the National Center for Global Health and Medicine, and Gunma University. Participants will undergo 4 weeks of treatment with acotiamide 300 mg/day, acotiamide 600 mg/day, or a placebo. Then, participants without remission of symptoms will undergo another 4 weeks of treatment with acotiamide 600 mg/day. Forty-two patients will be recruited and followed up for a maximum of 10 weeks. The study protocol was written in accordance with the Standard Protocol Items: Recommendations for Interventional Trials (SPIRIT) statement and approved by the Institutional Review Board (IRB) of Kyushu University for Clinical Trials (IRB No. 2021301; June 28, 2021). The trial was registered in the Japan Registry of Clinical Trials (jRCT; No. jRCT2071210072: October 11, 2021) before patient enrollment. All patients participating in the study will give written consent obtained by the researchers. The flow of the study is illustrated in Fig. 1.

### Eligibility criteria {10}

#### Inclusion criteria

The study population will include patients with clinically diagnosed idiopathic EGJOO at study site institutions.

The inclusion criteria will be as follows:

1. Patients with the age of 20 or over at the time of consent.
2. Patients diagnosed with EGJOO by HRM according to Chicago Classification ver 3.0.
3. Patients who have been experiencing a sensation of food sticking in the chest for at least 1 month prior to the time of consent and have a score of 2 (“symptomatic and slightly troubled”) or higher on at least one evaluation of food-sticking symptoms during the screening period.
4. Patients who underwent esophagogastroduodenoscopy and esophagography and in whom other organic diseases that may cause symptoms (e.g., reflux esophagitis, eosinophilic esophagitis, gastric ulcer) were excluded.
5. Patients who understand the contents of the clinical trial and have given written consent of their own free will.

#### Exclusion criteria

Patients meeting any of the following criteria will be excluded:

1. Patients with clinically evident hepatic dysfunction (in whom aspartate transaminase or alanine transaminase levels in the screening period was three times or more than the upper limit of the institutional standard range).
2. Patients with severe renal dysfunction or renal insufficiency (creatinine clearance of less than 30 mL/min in the screening period).
3. Patients with a history of hypersensitivity to any component of the investigational drug.
4. Patients who took acotiamide orally during the month before obtaining consent.
5. Patients with concomitant malignancy. However, patients with completely resected basal cell carcinoma, stage I spinous cell carcinoma, intraepithelial carcinoma, intramucosal carcinoma, or superficial bladder cancer can be included.
6. Patients with frequent irregular dietary habits, such as not eating or binge drinking/eating.
7. Patients with a history of upper gastrointestinal surgery. However, patients who underwent surgeries related to endoscopic treatment, including polypectomy, mucosal resection, and submucosal dissection, will be excluded.
8. Patients who have participated in other clinical trials or clinical studies and used or are using investigational drugs, devices, or products during the 3 months before obtaining consent.
9. Patients with serious cardiac or hematological diseases.
10. Patients with serious drug allergies.
11. Female patients who are pregnant, lactating, or of childbearing potential.
12. Patients who refuse to use appropriate contraceptive methods during the study period.
13. Patients who are judged by the PI or sub-investigator to be ineligible for the study.

### Who will take informed consent? {26a}

Informed consent will be obtained by the PI and sub-investigator using the consent document and consent form before enrollment in the trial. When explaining the trial, the PI or sub-investigator must allow patients to ask questions and provide sufficient time to decide whether to participate in the trial before obtaining consent. Furthermore, investigators or collaborators providing supplemental explanations must answer all questions regarding patient satisfaction.

### Additional consent provisions for collection and use of participant data and biological specimens {26b}

Concerning the collection and use of participant data and biological specimens, possibilities of the secondary use of data and biological specimens will be mentioned in the same consent document as the trial description, which will be explained when signing the consent form. The participant will be deemed to have consented to secondary use as well.

## Interventions

### Explanation for the choice of comparators {6b}

A placebo was used as a comparator in this clinical trial. Administration of a placebo to patients in the control group (*n* = 14) is ethically justifiable because no drug is effective for patients with EGJOO in Japan.

The creation of a placebo intervention is essential for the successful blinding of the participants and research staff. Therefore, Zeria was requested to produce film-coated tablets without acotiamide as placebo tablets. In the placebo group, the placebo will be administered three times a day with two tablets per dose before meals for 4 weeks.

### Intervention description {11a}

#### Pre-treatment observational period

Participants who consented will be examined and observed to confirm their eligibility during the pretreatment observational period. The maximum duration of the pretreatment observational period will be 28 days after obtaining consent. During this period, participants will complete a “symptom diary” for seven consecutive days out of 10 before enrollment. If any of the drugs listed in the concomitant use prohibition had been used, their use will be discontinued after consent has been obtained. After confirmation of eligibility, the participants will be enrolled and randomized 1:1:1 to receive acotiamide 300 mg/day, acotiamide 600 mg/day, or a placebo.

#### Treatment period 1

Participants will take the assigned drugs three times per day before meals for 4 weeks and complete the “application of Medication and Symptom Diary.” Participants will visit the institution after 4 weeks of treatment in period 1 (day 29; validity period: days 28–32) to undergo pre-defined examinations and observations, including HRM and symptom assessment.

#### Treatment period 2

Participants who complete treatment period 1 will take 200 mg/dose of acotiamide three times per day before meals for 4 weeks in an open-label fashion and complete the “application of Medication and Symptom Diary.” Patients will visit the institution after 4 weeks of treatment in period 2 (day 29; validity period: days 28–32) to undergo pre-defined examinations and observations, including HRM and symptom assessment.

However, if IRP is normalized (less than the standard value of IRP set for each HRM instrument) based on the HRM study and the worst value of the symptom of food sticking in the chest is 0 (elimination case) after 4 weeks of treatment in period 1 (day 29; validity period: days 28–32), participants will not proceed to treatment period 2 but will transfer to the post-treatment observational period.

#### Post-treatment observational period

Participants who complete the treatment periods will record an “application of Medication and Symptom Diary” for 14 days after completion of medication. Participants will visit the institution after the 2-week post-treatment observation period (days 15–18) to undergo pre-defined examinations and observations, including symptom assessment.

### Criteria for discontinuing or modifying allocated interventions {11b}

In any of the following cases, the clinical trial for the participants will be discontinued:

1. If investigators conclude that the continuation of the study will be difficult owing to the worsening of the primary disease (such as progression to achalasia).
2. If investigators conclude that the continuation of the study will be difficult because of the occurrence of adverse events.
3. If participants request the discontinuation of the clinical trial or withdraw consent.
4. If performing follow-ups with participants will become difficult owing to reasons such as patients no longer visiting the institution.
5. If the following serious deviations from the study protocol are revealed:
  If participants’ ineligibility is discovered after enrollment.
  If participants require or continue the administration of a prohibited concomitant drug.
6. If pregnancy is detected.
7. If investigators conclude that the continuation of the clinical trial will be difficult because of other reasons.

### Strategies to improve adherence to interventions {11c}

To improve adherence to the interventions, the dosing regimen will be simplified, with the same number of tablets and timing of all doses. Before dosing, investigators will instruct patients when to take the medication and what to do if they forget to take it.

1. The investigational drug should be taken before breakfast on the day after the initial prescription.
2. Two tablets of the investigational drug should be taken three times per day before each meal.
3. The dosage of the investigational drug should not be changed based on the patient’s judgment.
4. Medication status (data, time, dosage, and the reason for missed doses) should be recorded in the “application of Medication and Symptom Diary.”
5. Missed doses and the used press-through-package sheet must be kept and brought to the next visit.

The dosing status will be collected using an electronic patient diary, and daily alerts will be set up for patient diary entries.

### Relevant concomitant care permitted or prohibited during the trial {11d}

After obtaining consent, the use of the following drugs will be prohibited until the completion or discontinuation of the clinical trial.

1. Gastroprokinetic agents: mosapride, domperidone, Rikkunshito (Kampo medicine; traditional Japanese medicine), metoclopramide, and itopride.
2. Other investigational drugs.
3. Acotiamide (as a non-investigational drug).
4. Choline stimulants: acetylcholine chloride, carpronium chloride, bethanechol chloride, and aclatonium napadicylate.
5. Cholinesterase inhibitors: donepezil hydrochloride, ambenonium chloride, distigmine bromide, pyridostigmine bromide, and neostigmine.

Endoscopic examinations, except upper gastrointestinal endoscopy during the observational period and gastrointestinal angiography, will be prohibited during the study period.

### Provisions for post-trial care {30}

Participants will not be allowed to continue receiving the investigational drug after the completion of this study. After participation in this study, another treatment deemed optimal for the patient will be selected and provided.

### Outcomes {12}

#### Primary endpoint measurement

The primary endpoint is the proportion of patients exhibiting an improvement in symptoms of food sticking in the chest after 4 weeks of treatment period 1.

“Looking back on your day, did you have any troubles with symptoms of food sticking when you swallowed?” Patients will respond to the question on the following six levels:

- 0: No symptoms
- 1: I had symptoms but had no troubles.
- 2: I had symptoms and had some troubles.
- 3: I had symptoms and was moderately troubled.
- 4: I had symptoms and was quite troubled.
- 5: I had symptoms and was extremely troubled.

The percentage of patients showing an improvement in symptoms is defined as the percentage of patients whose worst symptom score in the latest week was 0 (no symptoms) or 1 (symptoms but no troubles) out of the six levels and whose score improved by at least two points.

#### Secondary outcomes measurement

Follow-up will be performed after 4 weeks of treatment period 1 as a secondary outcome measure.

1. The proportion of patients with normalized IRP on HRM.
2. The value of change from baseline in parameters, including distal contractile integral (DCI) and basal LES pressure (BLESP) on HRM.
3. The value of change from the baseline in the symptom of food sticking in the chest.
4. The value of change from baseline in EGJOO–QOL score (total and each score).
5. The value of change from the baseline in GSRS.
6. The correlation between IRP and each symptom score.

Furthermore, exploratory evaluations after 4 weeks of treatment period 2 will be conducted as a secondary outcome.

1. The proportion of patients showing improvement and the value of change from baseline in symptoms of food sticking in the chest.
2. The value of change from baseline and proportion of patients with normalized IRP on HRM.
3. The value of change from baseline in the EGJOO–QOL score (total score and each score).
4. The value of change from the baseline in GSRS (total and subscale scores).
5. The value of change from the baseline in DCI and BLESP measured by HRM.

Furthermore, the proportion of participants reporting the recurrence of symptoms of food sticking in the chest after 2 weeks of the post-treatment observational period will be assessed.

### Participant timeline {13}

**Fig. 2:**
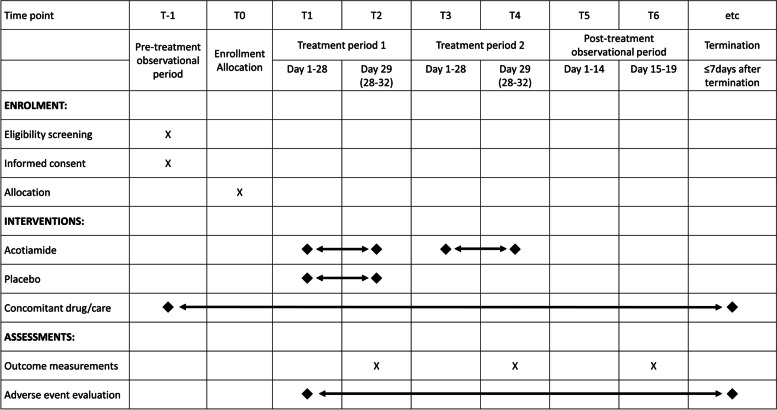
SPIRIT flow diagram

The protocol conforms to the SPIRIT guidelines (Supplemental File 1). The participant timeline during the trial is shown in Fig. 2.

### Sample size {14}

The primary analysis will be a comparison of treatment outcomes between the acotiamide (the 300 mg/day and 600 mg/day groups will be pooled) and placebo groups. The secondary analysis will be a comparison of treatment outcomes between the 300 mg/day and 600 mg/day groups to investigate the dose–response to acotiamide. Therefore, the sample size will be set based on a comparison between the acotiamide and placebo groups (allocation ratio 2:1).

In the prospective observational study conducted at Kyushu University Hospital, 10 out of 23 participants (43.5%), who answered the upper abdominal symptom questionnaire (Revised F Scale) after 4 weeks of acotiamide administration (300 mg/day), answered “No” to “Do you feel any symptom of food sticking in the chest (grabbing) when you swallow something?” as a question of the symptoms of food sticking in the chest. Although a scale different from the Revised F Scale will be used to evaluate the efficacy of acotiamide in the current trial, we expect that a similar proportion of patients will show an improvement in symptoms of food sticking in the chest after acotiamide administration. Therefore, we assume that 55% of patients in the acotiamide group will show an improvement in the symptom of food sticking in the chest. In contrast, 15% of patients in the placebo group are assumed to show an improvement in their symptoms. Considering the exploratory nature of this clinical trial, 39 patients are needed to reach a power of 80% for a normal approximation to the binomial distribution with a one-sided significance level of 5%. Considering the possibility of one dropout per group, the target number of participants is set to 42 (14 in each group).

### Recruitment {15}

Identification, screening, and consent procedures will be undertaken by the PI, sub-investigator, and collaborator who will be trained and competent to participate according to the ethically approved protocol, principles of Good Clinical Practice (GCP), and the Declaration of Helsinki. We implanted a multi-faceted approach to ensure an adequate enrollment of participants to reach our target sample size. First, we displayed informational posters in participating institutions to enhance the patient awareness of the trial. Second, we held lectures for local family physicians to explain the nature and objectives of the trial. Last, we conducted public lectures to educate the general public about EGJOO and the specifics of this trial. This comprehensive strategy was designed to facilitate a broad understanding and encourage enrollment in the trial. The recruitment period has been designed for 2 years. The number of eligible patients per institution in a month is estimated to be approximately one. Assuming a 50% consent rate, the enrollment is expected to be completed in 2 years.

## Assignment of interventions: allocation

### Sequence generation {16a}

The allocation manager will prepare the allocation chart of investigational drugs (300 mg/day group, 600 mg/day group, or placebo group) and randomly assign participants to each group with a 1:1:1 allocation using the substitution block method stratified by institution. The allocation manager assigns participants to each group randomly using a computer-generated allocation table for the investigational drug (300 mg/day group, 600 mg/day group, or placebo group), employing a stratified blocked randomization method stratified by the institution, with a 1:1:1 allocation ratio.

### Allocation concealment mechanism {16b}

The allocation manager will conceal the allocation chart immediately after the completion of the allocation and keep it in a sealed envelope until the key is opened. In addition, the manager will prepare the emergency key code, which is stored and managed in the electronic data capture (EDC) system. The key code should not be opened except in accordance with the predetermined procedure.

### Implementation {16c}

The PIs or sub-investigators will confirm that the participants meet all the inclusion criteria and do not meet any of the exclusion criteria. The investigators will then obtain the identification code for the participants and register the participants using a web-based registration system. Upon the completion of registration, “eligible” participants will be assigned a drug number. The investigators will prescribe the investigational drug with the drug number obtained from the web registration system to that participant. The study will be terminated and appropriate measures will be taken for “non-eligible” participants.

## Assignment of interventions: blinding

### Who will be blinded? {17a}

This is a double-blind trial. Both the research staff and participants will be blinded. All data will be managed, evaluated, and statistically processed by a third party following the clinical research. Outcome evaluators will not be informed of the group of belonging. Data analysts will work on the final dataset where the group condition will be masked.

### Procedure for unblinding if needed {17b}

The PIs and sub-investigators may request the coordinating investigator to open the emergency key code if deemed necessary to identify the dose groups of the investigational drug to treat a participant in a medical emergency, such as the occurrence of a serious adverse event. Upon receiving the request, the coordinating investigator will consult with the allocation manager as necessary and determine the measures. If the coordinating investigator decides to open the code, the coordinating investigator will immediately request that the allocation manager open the code and receive a report on the dose group to which the investigational drug has been allocated. If possible, the PI or sub-investigator will send a report about the safety and efficacy of the investigational drug before opening the code. The coordinating investigator will then report the result of opening the code to the PI or sub-investigator. The coordinating investigator will record and maintain the process.

## Data collection and management

### Plans for assessment and collection of outcomes {18a}

Data will be collected at the following time points:

- T-1: Pre-enrollment observation period: seven consecutive days within 10 days prior to enrollment
- T0: Enrollment
- T1: During treatment period 1: days 1–28 of treatment period 1
- T2: After 4 weeks of treatment period 1 (scheduled visit): days 29–32 of treatment period 1
- T3: During treatment period 2: days 1–28 of treatment period 2
- T4: After 4 weeks of treatment period 2 (scheduled visit): days 29–32 of treatment period 2
- T5: During the post-treatment observational period: days 1–14 of the post-treatment observational period
- T6: After 2 weeks of the post-treatment observational period (scheduled visit): days 15–19 of the post-observational treatment period

Patient symptoms and medication status will be assessed using the electronic patient-reported outcomes (ePRO) system. Patients will download a specialized patient diary app on their smartphones or tablets and respond to the questions. ePRO data will be directly transmitted to the EDC system and can be monitored at any time.

### Plans to promote participant retention and complete follow-up {18b}

As part of the plan to facilitate the retention of participants and full follow-up with patients, the following will be implemented: the significance of the trial and medication compliance will be fully explained, and participants will be asked to come to the hospital according to the study schedule. To reduce the burden on participants, past test results will be made available as part of screening procedures. Payment for cooperation in clinical trials will also be offered to reduce the burden of transportation and other expenses. ePRO will allow participants to enter their “application of Medication and Symptom Diary” using an easy-to-use app on their devices and set reminders on the app for them to enter their logs daily.

### Data management {19}

Investigators will promptly prepare or revise the case report form (CRF) using the EDC system following the input manual. The data described in the CRF must be consistent with data from source documents. If any discrepancy between the source document and CRF exists, the reason for the discrepancy must be explained. Investigators must ensure that the CRF data are accurate and complete. The PI will confirm the adequacy of the prepared CRF content and sign it electronically. Data are stored securely in the study databases and only accessible to research personnel (e.g., investigators, CRCs, monitors, and data managers) trained in confidentiality and privacy procedures.

### Confidentiality {27}

All parties involved should give sufficient consideration to the protection of patients’ personal information and privacy, in accordance with relevant laws and regulations. When investigators report data related to the trial, such as CRFs, patients should be identified by the identification code instead of their names and medical record numbers. When monitors, auditors, clinical trial review committees, and regulatory authorities have direct access to source documents, patients’ privacy should be preserved. Similarly, when disclosing the results of the trial, the patients’ privacy should be preserved. Furthermore, the information obtained in the trial should not be leaked to any third party, except when requested by public authorities.

### Plans for collection, laboratory evaluation, and storage of biological specimens for genetic or molecular analysis in this trial/future use {33}

N/A, as biological data for genetic or molecular analyses will not be collected.

## Statistical methods

### Statistical analysis methods for primary and secondary outcomes {20a}

The analysis of the primary endpoint will be conducted in the full analysis set, which will include all randomized patients who received at least one dose of treatment during the study, data collected after treatment commencement, and adherence to GCP. A one-sided *p*-value < 0.05 will be considered statistically significant. The following closed testing procedure will be used to compare the three groups: the placebo group as the control group, the group with 300 mg/day acotiamide, and the group with 600 mg/day acotiamide.

1. The placebo and acotiamide groups (300 mg/day and 600 mg/day groups will be pooled) will be analyzed using a normal approximation to the binomial distribution test. If the difference is significant, the next step will be followed. Otherwise, the procedure will be terminated.
2. For the acotiamide groups (300 mg/day and 600 mg/day), a normal approximation to the binomial distribution test will be performed.

In addition, regardless of whether significance is observed in 1 and 2 above, the difference in the proportion of patients who improve between the placebo and acotiamide groups and between the two acotiamide groups, and the two-sided 90% confidence interval will be evaluated.

As secondary endpoints are exploratory endpoints, proportions will be analyzed using a normal approximation to the binomial distribution test with the placebo and acotiamide groups. Similarly, the same method will be used to test the 300 mg/day group against the 600 mg/day group. The change from the baseline will be analyzed using analysis of covariance (ANCOVA) with the baseline values, as covariates will be employed to test with the placebo and acotiamide groups. Furthermore, the same method will be applied to test the superiority of the 600 mg/day group over the 300 mg/day group.

SAS software (version 9.4; SAS Institute, Cary, NC, USA) will be used for all analyses.

### Interim analyses {21b}

No interim analysis is planned.

### Methods for additional analyses (e.g., subgroup analyses) {20b}

The subgroup analyses will be performed exploratively using a range of variables.

### Methods in analysis to handle protocol non-adherence and any statistical methods to handle missing data {20c}

For the primary endpoint, missing values for any evaluation of the 7-day at 4 weeks of treatment period 1 will be imputed with the average value for that week. Patients with more than 4 days of missing data in a week will be excluded from the analysis.

### Plans for giving access to the full protocol, participant-level data, and statistical code {31c}

The full protocol, de-identified datasets, and statistical code are available from the corresponding author upon reasonable request.

## Oversight and monitoring

### Composition of the coordinating center and steering committee {5d}

The steering committee has been established by the principal investigators (PIs), coordinating investigators, and study managers. Study managers have been constituted by the Academic Research Organization (ARO) of Kyushu University.

Cmic Co., Ltd. will independently conduct a study on drug allocation, data management, monitoring, and auditing through a contract as an external company that specializes in clinical trial activities. This study will be monitored to ensure adherence to ethical aspects, participants’ rights, and the quality of data documentation. Statistical analyses will be performed by statisticians at the ARO of Kyushu University. The corresponding author (EI) is the PI representative and project manager. Day-to-day support will be provided by EI and ARO.

The coordinating center has been established by the coordinating investigator and clinical research coordinators (CRCs), monitors, and study managers. The PIs will be responsible for the overall overview of the trial. The coordinating investigators and CRCs will provide day-to-day support for the trial and hold meetings once a month.

### Composition of the data monitoring committee, its role and reporting structure {21a}

Cmic Co., Ltd. will monitor the trial to ensure that the investigational team complies with the study protocol and GCP standards, the data and adverse events (AEs) are accurately and appropriately recorded in the eCRFs, severe AEs (SAEs) are forwarded to the trial coordinator and the investigational drug provider, and SAEs meeting reporting criteria are forwarded to the IRB. During the study, the PI will meet with each municipality to monitor participant safety and data assessment procedures. The coordinating center will organize monthly meetings to review the trial conduct.

### Adverse event reporting and harms {22}

When an SAE occurs, the PI or sub-investigators will promptly take proper measures to ensure patient safety. They must immediately report the SAE to the head of the institution and coordinating investigator in accordance with the “Procedures for Handling Safety Information” for the relevant clinical trial. The coordinating investigator will immediately submit a report to the other PIs and the provider of the investigational drug and will evaluate and discuss the SAE and how to respond to the SAE with all PIs. If the reported SAE meets the criteria of enforcement regulations in Article 273 of the Pharmaceuticals and Medical Devices Law, the report will be submitted to the Pharmaceuticals and Medical Devices Agency and the details will be reported to the head of the institution, PIs, and the provider of the investigational drug. The PI will conduct follow-up investigations on all SAEs, and the obtained information will be promptly handled in the same manner as described above.

### Frequency and plans for auditing trial conduct [23]

The trial audit will be conducted by Cmic Co., Ltd. approximately once a year, according to the audit plan. The steering committee, monitors, and IRB will hold meetings to conduct a review once a month throughout the trial period.

### Plans for communicating important protocol amendments to relevant parties (e.g., trial participants, ethical committees) {25}

Any further necessary protocol amendments will be communicated timeously to the IRB of each institution. A revised copy will be stored, and the protocol in the clinical trial registry will be updated. Any amendments or changes will also be described transparently in publications following the trial. Participants will also be informed orally or in writing of any amendments to the protocol.

### Dissemination plans {31a}

Trial results will be published in peer-reviewed journals of general and special interest and presented at international conferences. Authorship and the degree of involvement will follow the Vancouver guidelines. Furthermore, we will write Japanese reports and present the results on web pages and social media platforms.

## Discussion

Currently, no definitive treatment for EGJOO has been established. Acotiamide is expected to improve LES accommodation in patients with EGJOO, similar to the accommodation of the gastric body and fundus seen in patients with FD. Indeed, the prospective observational clinical studies have reported that acotiamide improves impaired accommodation and normalizes IRP levels in 80% and 52% of patients with EGJOO [8]. This study was designed based on the previous observational studies and is the first prospective comparison study to evaluate the efficacy and the safety of acotiamide in patients with EGJOO. We predict that a significant difference in outcomes between the acotiamide and control groups will be observed; this will assist the drug repositioning of acotiamide for EGJOO. Acotiamide will contribute to improving the QOL of patients with EGJOO and is expected to prevent the progression of EGJOO to achalasia.

## Trial status

The recruitment for this trial began on October 15, 2021. The first participant was recruited on December 13, 2021. The trial is ongoing. The recruitment period will end on September 30, 2023. The study protocol data: Initial approval June 28, 2021; Current version (4.0) April 22, 2022.

## Supplementary Information

**Additional file 1.** SPIRIT 2013 Checklist: Recommended items to address in a clinical trial protocol and related documents*.

## Data Availability

Only the PI, local co-investigator, and research staff at ARO will have access to the final dataset solely for research purposes. The datasets generated and analyzed during the current study will not be made publicly available to protect participant privacy but are available from the corresponding author upon reasonable request.

## Abbreviations

AE: Adverse event
AMED: Agency for Medical Research and Development
BLESP: Basal lower esophageal sphincter pressure
CRC: Clinical research coordinator
CRF: Case report form
DCI: Distal contractile integral
EDC: Electronic data capture
EGJOO: Esophagogastric junction outflow obstruction
EGJOO–QOL: Esophagogastric junction outflow obstruction–quality of life
EMD: Esophageal motility disorder
ePRO: Electronic patient-reported outcomes
FD: Functional dyspepsia
GCP: Good Clinical Practice
GSRS: Gastrointestinal Symptom Rating Scale
HRM: High-resolution manometry
IRB: Institutional review board
LES: Lower esophageal sphincter
PI: Principal investigator
SAE: Severe adverse event
SPIRIT: Standard Protocol Items: Recommendations for Interventional Trials

## References

[1] Kahrilas PJ, Bredenoord AJ, Fox M, Gyawali CP, Roman S, Smout AJ, Pandolfino JE, International High Resolution Manometry Working G (2015). The Chicago Classification of esophageal motility disorders, v3.0. Neurogastroenterol Motil.

[2] Yadlapati R, Kahrilas PJ, Fox MR, Bredenoord AJ, PrakashGyawali C, Roman S, Babaei A, Mittal RK, Rommel N, Savarino E (2021). Esophageal motility disorders on high-resolution manometry: Chicago classification version 4.0((c)). Neurogastroenterol Motil.

[3] Kahrilas PJ, Boeckxstaens G (2013). The spectrum of achalasia: lessons from studies of pathophysiology and high-resolution manometry. Gastroenterology.

[4] Muta K, Ihara E, Hamada S, Ikeda H, Wada M, Hata Y, Bai X, Nishihara Y, Tanaka Y, Ogino H (2021). Physiological and pathological roles of the accommodation response in lower esophageal sphincter relaxation during wet swallows. Sci Rep.

[5] Trifan A, Shaker R, Ren J, Mittal RK, Saeian K, Dua K, Kusano M (1995). Inhibition of resting lower esophageal sphincter pressure by pharyngeal water stimulation in humans. Gastroenterology.

[6] Nakajima T, Nawata H, Ito Y (2000). Z-338, a newly synthetized carboxyamide derivative, stimulates gastric motility through enhancing the excitatory neurotransmission. J Smooth Muscle Res.

[7] Matsueda K, Hongo M, Tack J, Saito Y, Kato H (2012). A placebo-controlled trial of acotiamide for meal-related symptoms of functional dyspepsia. Gut.

[8] Ihara E, Ogino H, Muta K, Hamada S, Wada M, Hata Y, Ikeda H, Bai X, Minoda Y, Esaki M (2022). The treatment effects of acotiamide in esophagogastric outflow obstruction: a prospective longitudinal observational study. Esophagus.

[9] Muta K, Ihara E, Fukaura K, Tsuchida O, Ochiai T, Nakamura K (2016). Effects of acotiamide on the esophageal motility function in patients with esophageal motility disorders: a pilot study. Digestion.

